# Technique notes on the management of superior sagittal or transverse sinus during the falcotentorial meningioma surgery: a case report

**DOI:** 10.3389/fneur.2024.1284038

**Published:** 2024-05-30

**Authors:** Jun Liu, Di Fan, Ligang Chen, Zheng Zou, Xinning Li, Minghao Zhou, Zhongcheng Wen, Shun Gong, Guobiao Liang

**Affiliations:** Department of Neurosurgery, General Hospital of Northern Theater Command, Shenyang, China

**Keywords:** meningioma, approach, confluence sinuses, technique note, venous bleeding

## Abstract

**Background:**

Falcotentorial meningiomas (FM) are surgical challenges for protecting sinus, and the technique notes on the management of superior sagittal or transverse sinus are required for good results.

**Methods:**

We improved the technique notes on the management of superior sagittal or transverse sinus in three FM patients with signs of increased intracranial pressure or chronic headache.

**Results:**

All patients underwent surgeries in the prone position, and occipital/sup-occipital/sub-occipital craniotomy was performed. In one patient, the skull was removed traditionally with exposure of the confluence of sinuses, superior sagittal, and transverse sinus, while the longitudinal skull bridge was left to suspend the dura for protecting the superior sagittal sinus in one patient, and the transverse skull bridge was left to suspend the dura for protecting the transverse sinus in one patient. The dura was opened infratentorially or supratentorially to spare the sinus and then the “skull bridge” was suspended. The tumor was then removed completely without brain swelling or significant venous bleeding. Complete tumor resection was confirmed by early postoperative imaging, and all patients recovered well without postoperative morbidity.

**Conclusion:**

The authors recommend the “skull bridge” to suspend the dura for optimal control of the venous sinuses during FM surgery (less venous bleeding).

## Introduction

Falcotentorial meningiomas (FM) are relatively rare tumors that arise from the meninges (the protective coverings of the brain and spinal cord), representing only 2%−3% of all intracranial meningiomas. These particular meningiomas are located at the junction between the tentorium (a membrane separating the cerebrum from the cerebellum) and the falx (a vertical fold within the skull). Due to their anatomical location, FM can present with a wide range of symptoms such as headaches, seizures, visual disturbances, and motor deficits (1). Treatment usually involves surgical resection, although adjuvant therapies such as radiation may be considered depending on the tumor size and location. Meningiomas, in general, are typically slow-growing tumors that tend to be benign but can occasionally cause significant neurological complications if left untreated. Therefore, early detection and intervention are crucial in managing these challenging lesions (2).

Complications related to the surgical approach to FM can be significant and challenging. Due to the location of these tumors near critical structures such as the deep venous sinuses and cranial nerves, there is a high risk of neurological deficits postoperatively. Intraoperative bleeding and damage to the surrounding vasculature are also common concerns. Additionally, due to their proximity to the tentorium and the complexities involved in achieving adequate surgical exposure while minimizing trauma to surrounding structures, complete tumor removal can be difficult to achieve without compensating crucial neurological function (3, 4). Close monitoring of patient's neurological status during surgery is essential to detect any potential complications early on and prevent long-term disabilities.

FM with supra- and infra-tentorial expansion need to be treated with distinct surgical strategies due to their anatomical relation to the venous sinuses (5, 6). While 7%−10% of meningiomas are located in the posterior fossa, only approximately 1% of these invade or occlude the confluence of sinuses (7). Given their rarity and the treatment in this region, not many neurosurgeons have extensive surgical experience with falcotentorial meningiomas involving the confluence of sinuses (8). Apart from the patient's position, the meningiomas' location, the extension of craniotomy, and the dura opening, the technique notes for protecting the confluence of sinuses, superior sagittal, and transverse sinus should be paid particular attention to achieve complete resection with less venous bleeding (9, 10).

## Case description

**Figure 1:**
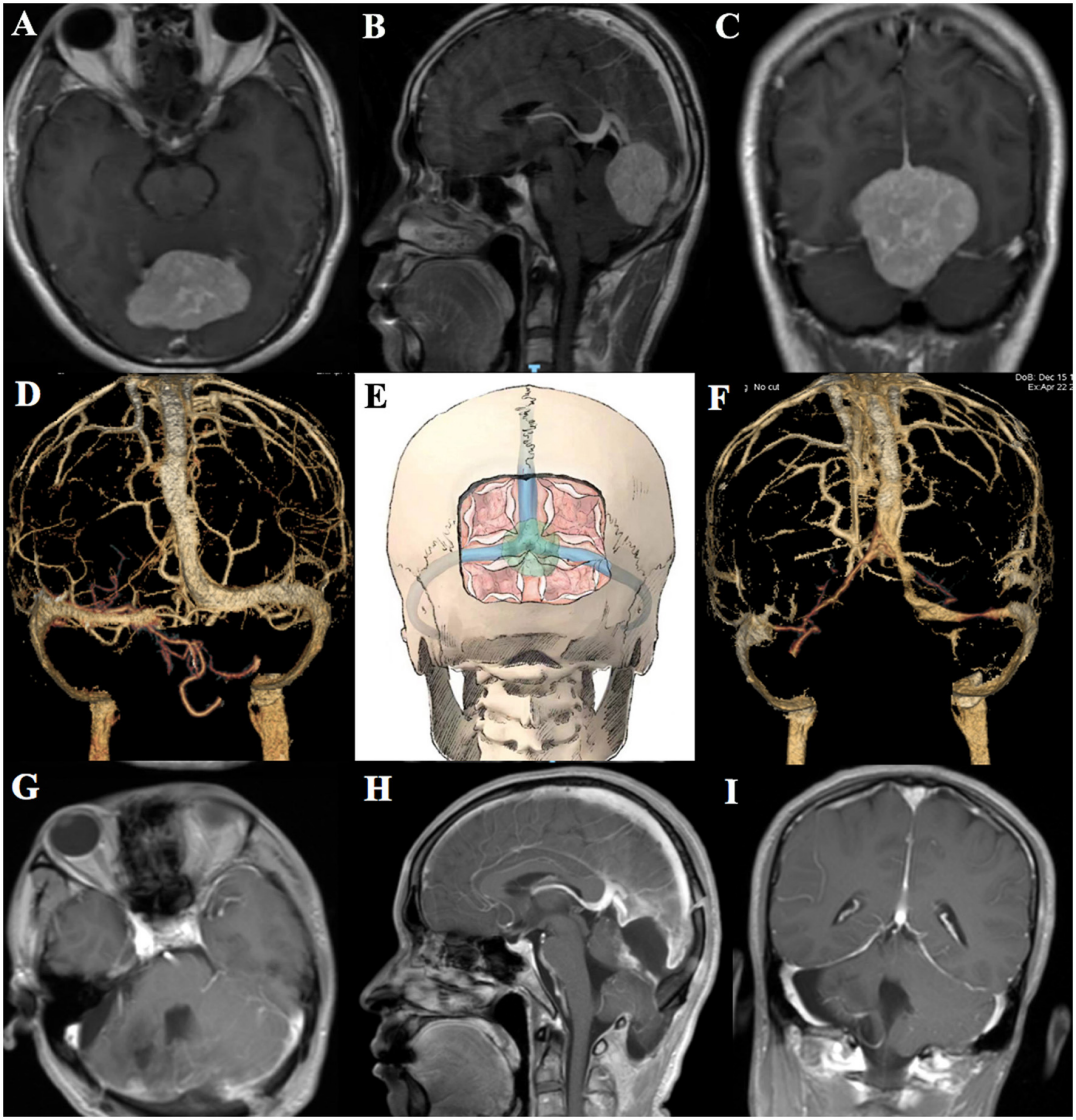
Preoperative axial, coronal, sagittal MRI, and CTV of patient 1 **(A–D)**. Schematic diagram of the extension of craniotomy and the dura opening **(E)**. Postoperative CTV and axial, coronal, and sagittal MRI of patient 1 **(F–I)**.

Patient 1 (a 40-year-old man) suffered from nausea, vomiting, visual disorders, and other signs of increased intracranial pressure. He underwent cerebral enhanced magnetic resonance imaging (MRI), which revealed that the tumor size was 5.2 × 4.1 × 3.8 cm (Figures 1A–C). The preoperative CT venography (CTV) indicated patency of venous sinuses (Figure 1D). Then, the operation was performed with the patient lying in a prone position, and an occipital and sub-occipital craniotomy was done to expose the confluence of sinuses, superior sagittal, and transverse sinus (Figure 1E). The dura was opened supratentorially to reduce the blood supply, and the gelatin sponge and cotton tape were used to stop the sinuum bleeding, but it is more difficult than ever to stop bleeding. Then, the major part of the meningioma was resected infratentorially to release the compression of the cerebellum. The transverse sinus was opened after the removal of the meningioma and reconstructed by the autologous fascia. Postoperative enhanced MRI showed complete resection of the meningioma, but the patency of the venous sinus in the confluence of sinues area was somewhat affected (Figures 1F–I) and the patient had postoperative headache and received corresponding treatment.

**Figure 2:**
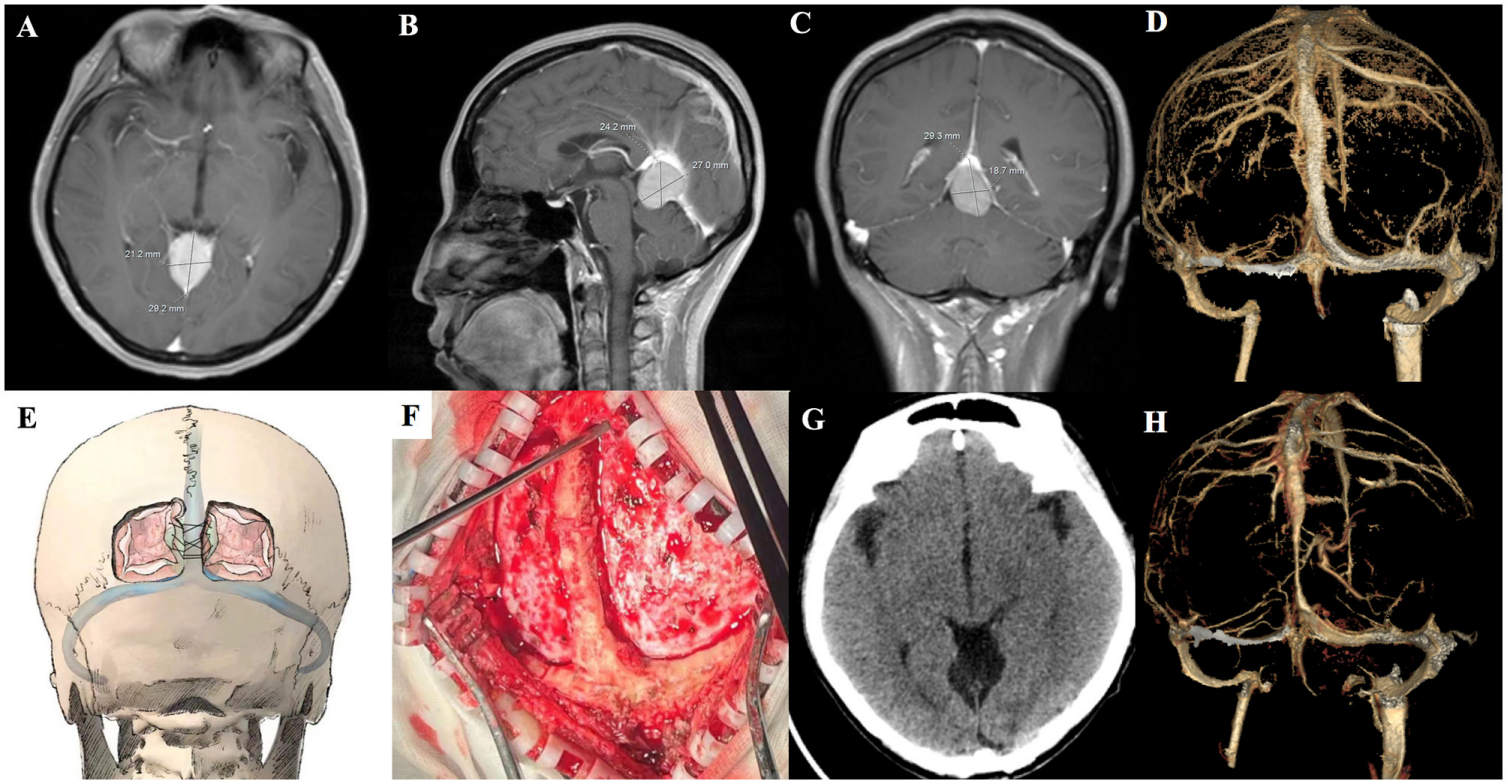
Preoperative axial, coronal, sagittal MRI, and CTV of patient 2 **(A–D)**. Schematic diagram of the extension of craniotomy and the dura suspension **(E)**. Intraoperative photo shows the extension of craniotomy **(F)**. Postoperative head CT/CTV of patient 2 **(G, H)**.

Patient 2 (a 39-year-old woman) had chronic headache and underwent cerebral enhanced MRI, which revealed that the FM size was 2.9 × 2.4 × 2.1 cm, and the tumor was close to the cerebral vein of Galen (Figures 2A–D). She was operated by laying her in a prone position, and the occipital craniotomy was performed to expose the superior sagittal sinus as well as the confluence of sinuses. Based on previous experience, to avoid venous sinus bleeding, we designed a longitudinal “skull bridge” to provide the support for suspending the dura to compress and stop bleeding of the superior sagittal sinus (Figures 2E, F and Supplementary Video 1). After suspending and opening the dura supratentorially parallel to the superior sagittal sinus, local decompression was achieved by cerebrospinal fluid (CSF) release. There was no brain swelling or venous bleeding, allowing the complete removal of the meningioma without deep cerebral drainage vein injury. Postoperative head CT/CTV showed complete resection of the tumor, and the venous sinuses was unobstructed (Figures 2G, H).

**Figure 3:**
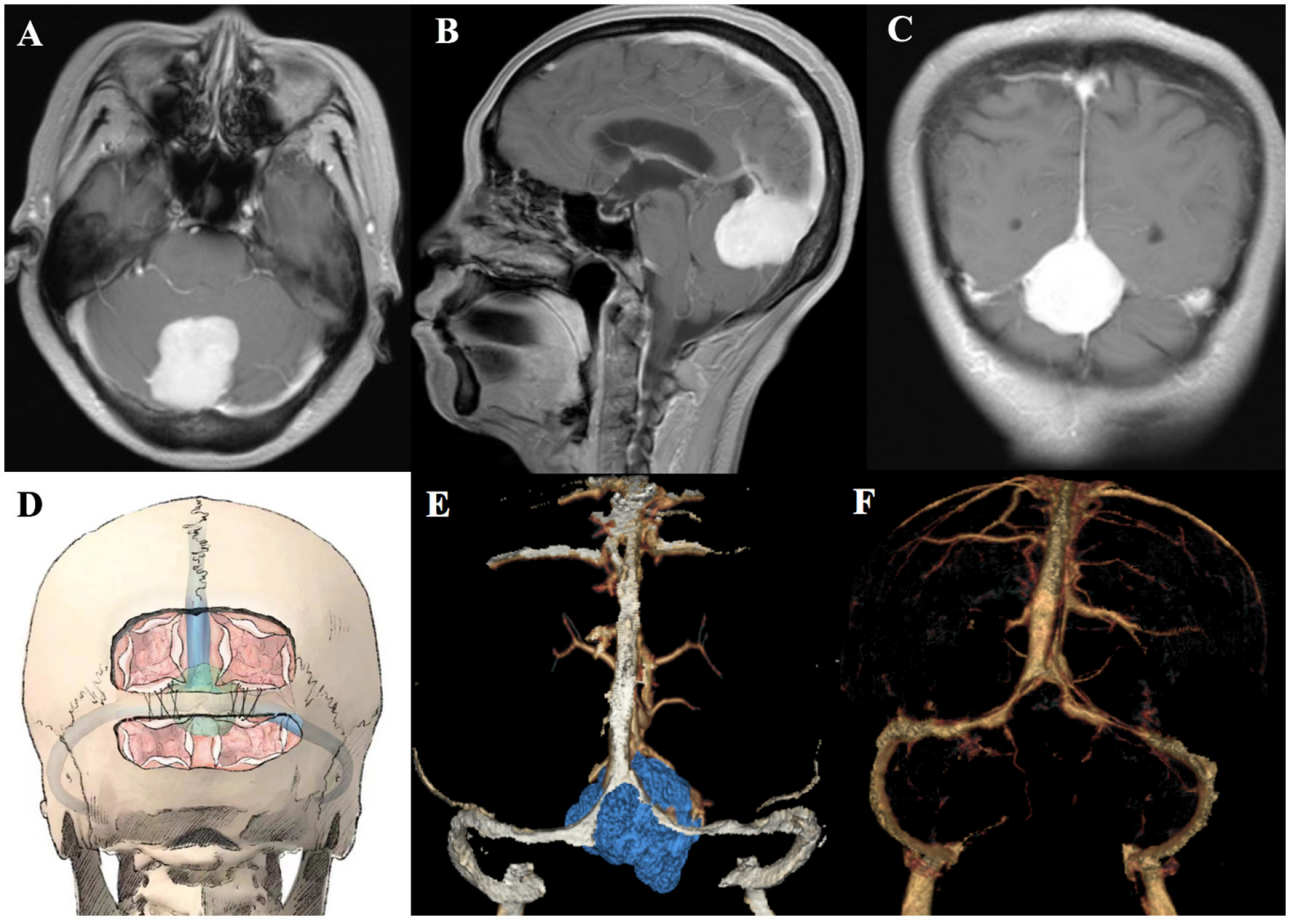
Preoperative axial, coronal, and sagittal MRI of patient 3 **(A–C)**. Schematic diagram of the extension of craniotomy and the dura suspension **(D)**. Preoperative and postoperative head CTV of patient 3 **(E, F)**.

Patient 3 (a 56-year-old woman) also suffered from chronic headache and underwent a cerebral enhanced MRI, which revealed the tumor size of 2.6 × 3.3 × 4.0 cm (Figures 3A–C). The surgical method was identical to that of patient 1 and patient 2. A transverse skull bridge was left to suspend the dura after an occipital and suboccipital craniotomy for protecting the transverse sinus (Figure 3D and Supplementary Video 2). Then, the dura was opened infratentorially and supratentorial parallel to the transverse sinus and along the superior sagittal sinus, allowing a perfect overview and access to the tumor. The combination of the infratentorial and supratentorial approach facilitated complete resection of the meningioma without damaging any venous sinus. Complete tumor resection, as well as patency of the sinuses, was confirmed by preoperative and postoperative head CT/CTV imaging (Figures 3E, F).

The intraoperative bleeding volume of three patients was 4,500, 1,000, and 800 mL, respectively. The durations of surgery, intensive care unit (ICU), and hospital stay of the patients are shown in Table 1. The histopathological examinations of all three patients demonstrated meningiomas of meningothelial type, and they have been discharged on postoperative days 7 to 9.

**Table 1:** Overview of the clinical data on the management of falcotentorial meningioma.

**Characteristic**	**Patient 1**	**Patient 2**	**Patient 3**
Age (years)	40	39	56
Sex	Male	Female	Female
Symptoms	Nausea, vomiting, visual disorders for 4 days	Chronic headache for 3 months	Chronic headache for 6 months
Comorbidity	Anemia	Uterine fibroids	Hypertension
Anesthesia method	General anesthesia,	General anesthesia	General anesthesia
Duration of surgery (hours)	11.5	8.6	8.2
Amount of bleeding (ml)	4,500	1,000	800
Duration of ICU (days)	5	3	2
Duration of hospital stay (days)	19	15	13

ICU, intensive care unit.

Postoperative follow-up of these patients was crucial in monitoring for any potential complications or recurrences following surgical intervention. Follow-up imaging at 1 year after resection showed no evidence of tumor recurrence. Neurological examinations of the patient's cognitive function, motor skills, and overall wellbeing at 1 year post-surgery were similar to the preoperative evaluation. Additionally, all three patients did not undergo any additional treatments such as radiation therapy. Overall, diligent postoperative follow-up care showed that these FM patients recovered well without any morbidity on follow-up at 1 year.

## Discussion

The FM involving the confluence of sinuses, superior sagittal, and transverse sinus have a wider spectrum of postoperative complications. Our cases are focused on those tumors with the potential risk of venous sinuses complications. The combination of occipital and sub-occipital craniotomy caused difficulty in stopping the bleeding of the sinus and affecting the patency of the venous sinus in the confluence of sinuses in one patient. However, the management of FM that invade the confluence of sinuses, superior sagittal, and transverse sinus is a subject of debate, particularly concerning the necessity of complete resection and protection of the venous sinus. Considering the structure of the sinus confluence area, to avoid the venous sinus damage in craniotomy, we reviewed articles and designed the “skull bridge” to suspend the dura for optimal control of the venous sinuses during the FM surgery that might expose the confluence of sinuses, superior sagittal, or transverse sinus.

The confluence of sinuses is the biggest obstacle when approaching the FM. A sufficient operative field is required by adequate sinus exposure, but sinus injury is a potentially life-threatening complication. The venous sinuses can be well-managed by utilizing different repair techniques with patency, but few studies have reported on how to protect them during craniotomy (11). Similar to our report, Gennaro Lapadula et al. presented two rare cases of epidural hematoma with dural sinus detachment and described how to firmly suspend the dura mater to the inner skull surface to prevent rebleeding (12). Unlike other surgical techniques that have been reported as the direct stitching, clipping, hitching up the dura to the bone adjacent to the sinus, or free and pedunculated duraplasty, our method of designing a “skull bridge” (the bony bridge upon the sinus) to suspend the dura being tied above the strip of bone has the advantages of being easier, faster, and providing a rigid structure to facilitate brain expansion and prevention of dural tear. Reducing intraoperative bleeding is crucial for safe removal of the FM involving the confluence of sinuses, superior sagittal, or transverse sinus. Therefore, we designed this “skull bridge” as a support point for intraoperative hemostasis to reduce sinus bleeding, shorten the surgical time, and achieve better prognosis for patients. However, during the tumor removal, the presence of the “skull bridge” slightly affected the visual field of the surgical area. This disadvantage could be compensated by adjusting the patient's position or the angle of the microscope. It would be more comfortable to perform such surgery through a combination of neuroendoscopy.

It is also reported that neuronavigation used for identifying the venous sinuses was very helpful in avoiding vascular injury during craniotomy (13). There was no FM in our cases showing any infiltration of the major venous sinus in the preoperative enhanced MRI. If such a situation was the case, the surgical strategy would have been different. Nasser Mohammed et al. recommended that the meningioma involving the sinus could be well-controlled by microsurgical techniques and adjuvant Gamma Knife radiosurgery with an acceptable complication rate (14). Nevertheless, we had one case with an opening of the transverse sinus and a significant swelling of the occipital lobe. Although it was controlled by lifting the head and applying autologous fascia for tissue sealing, it could be avoided by using a semi-sitting position and an infratentorial dural opening (15). In our opinion, a more significant cerebrospinal fluid drainage and venous drainage could effectively improve the approach to the FM and conveniently protect the venous sinuses.

## Conclusion

With our experiences, we prefer the “skull bridge” design to suspend the dura for optimal control of the venous sinuses during the FM surgery (less venous bleeding). Good cerebrospinal fluid drainage and brain relaxation should be combined with sinus sparing for adequate visualization and total meningioma removal.

## Data availability statement

The original contributions presented in the study are included in the article/Supplementary material, further inquiries can be directed to the corresponding authors.

## Ethics statement

The studies involving humans were approved by the local Ethics Committee of the General Hospital of Northern Theater Command. The studies were conducted in accordance with the local legislation and institutional requirements. The participants provided their written informed consent to participate in this study. Written informed consent was obtained from the individual(s) for the publication of any potentially identifiable images or data included in this article.

## Author contributions

JL: Writing—original draft, Visualization, Validation, Data curation, Conceptualization. DF: Writing—original draft, Visualization, Investigation, Conceptualization. LC: Writing—review & editing, Resources, Methodology, Investigation. ZZ: Writing—review & editing, Validation, Project administration, Investigation. XL: Writing—review & editing, Methodology, Investigation. MZ: Writing—review & editing, Investigation, Data curation. ZW: Writing—review & editing, Investigation, Data curation. SG: Writing—review & editing, Writing—original draft, Supervision, Methodology, Conceptualization. GL: Writing—review & editing, Visualization, Validation, Supervision, Resources, Funding acquisition, Formal analysis, Conceptualization.

## Supplementary material

Supplementary Video 1 The technique note of designing the longitudinal “skull bridge” to suspend the dura for optimal control of the venous sinuses during the falcotentorial meningioma surgery.

Supplementary Video 2 The technique note of designing the transverse “skull bridge” to suspend the dura for optimal control of the venous sinuses during the falcotentorial meningioma surgery.
